# Low literacy and written drug information: information-seeking, leaflet evaluation and preferences, and roles for images

**DOI:** 10.1007/s11096-016-0376-4

**Published:** 2016-09-21

**Authors:** Mara M. van Beusekom, Petronella Grootens-Wiegers, Mark J. W. Bos, Henk-Jan Guchelaar, Jos M. van den Broek

**Affiliations:** 1 Clinical Pharmacy and Toxicology, Leiden University Medical Center, Leiden, The Netherlands; 2 Science Communication and Society, Leiden University, Leiden, The Netherlands; 3 Communication, Faculty Management and Organisation, The Hague University of Applied Sciences, The Hague, The Netherlands

**Keywords:** Drug information, Literacy, Legibility, Netherlands, Patient information leaflet, Pictograms, Readability, Visuals

## Abstract

*Background* Low-literate patients are at risk to misinterpret written drug information. For the (co-) design of targeted patient information, it is key to involve this group in determining their communication barriers and information needs. *Objective* To gain insight into how people with low literacy use and evaluate written drug information, and to identify ways in which they feel the patient leaflet can be improved, and in particular how images could be used. *Setting* Food banks and an education institution for Dutch language training in the Netherlands. *Method* Semi-structured focus groups and individual interviews were held with low-literate participants (n = 45). The thematic framework approach was used for analysis to identify themes in the data. *Main outcome measure* Low-literate people’s experience with patient information leaflets, ideas for improvements, and perceptions on possible uses for visuals. *Results* Patient information leaflets were considered discouraging to use, and information difficult to find and understand. Many rely on alternative information sources. The leaflet should be shorter, and improved in terms of organisation, legibility and readability. Participants thought images could increase the leaflet’s appeal, help ask questions, provide an overview, help understand textual information, aid recall, reassure, and even lead to increased confidence, empowerment and feeling of safety. *Conclusion* Already at the stages of paying attention to the leaflet and maintaining interest in the message, low-literate patients experience barriers in the communication process through written drug information. Short, structured, visual/textual explanations can lower the motivational threshold to use the leaflet, improve understanding, and empower the low-literate target group.

## Impact of practice

- Building on the identified problems and proposed solutions presented by people with low-literacy allows for the development of a targeted, co-designed intervention to improve written drug information.
- Pharmacists should consider using images in written drug information that help to find and understand relevant topics and lower the motivational threshold to use the printed information.
- Images in patient leaflets can help to empower patients with low literacy.

## Introduction

Written drug information is generally difficult to read, causing patients to struggle with the interpretation of their medication instructions [1, 2]. This is a particular concern for patients with low literacy skills [3], who also out of the context of healthcare have difficulties with using printed and written information as encountered in society [4]. In this light, it is of little surprise that there is a relation between low (health) literacy levels and problems with safe use of medication and adherence to therapy [5, 6]. This is undesirable, as safe use of medication, including being observant of side-effects and multi-drug interactions, as well as proper adherence, is crucial for effective treatment and to advance patient health outcomes [7, 8].

Despite the described shortcoming, written drug information has important benefits compared to other information sources, also for people with low literacy. For example, patients generally have low retention of spoken information [9]. However, printed instructions, such as Patient Information Leaflets (PILs) that are packed with medicines, remain available for later reference and for relations of the patients, such as family and caretakers [10].

To improve the usability and quality of written drug information, visual aids can be used in conjunction with text to facilitate understanding—an approach that has proven to be particularly successful for people with low literacy [11–14]. To further illustrate, already in 1996, Delp and Jones [15] demonstrated that printed health instructions with visuals were much more likely to be read by patients than text-only materials. In addition to drawing attention, there is ample proof that images can aid free and cued recall [16–19].

This study explored the interaction between individuals with low literacy and patient information, with the intention to develop a visual/textual drug leaflet that is compatible with the needs a low-literate audience. Active involvement of the target group in the development process, or co-design, has been shown to lead to successful health interventions [20, 21]. To date, there is insufficient insight into the communication barriers and information needs of the low-literate target group, and there is a lack of studies that involve this audience in the design of targeted patient leaflets.

## Aim of the study

The aim of this study is to gain insight into how people with low literacy use and evaluate written drug information, and to identify ways in which they feel the patient leaflet can be improved, and in particular how images could be used.

## Ethics approval

Informed consent was obtained from all individual participants included in the study. Following national regulations, no ethical approval was required for this type of study.

## Method

Data were collected through individual interviews and focus group discussions with participants with low literacy, making use of a one-on-one approach to acquire detailed information and group interactions to allow participants to discuss and reach consensus. An iterative process allows the researcher to build upon obtained results, and triangulate data by identifying missing pieces of information and confirming recurring stories [22]. For example, if participants of the focus group discussions do not feel they can speak freely about personal issues in a group, this type of information could potentially be shared in the individual interviews.

### Theoretical approach

Following principles of human factors and ergonomics [23], the target group was involved in the ‘fuzzy front end’ of the design process. In this step of the design process it is determined what problems should be targeted and what the product should look like [24].

McGuire’s communication/persuasion matrix will be used to provide context to the interpretation of the results, as previously suggested for health-related images by Houts [25]. In his model, McGuire describes how people, before being persuaded by a (health-related) message, transit through 13 stages: tuning in, attending, liking, comprehending, generating, acquiring, agreeing, storing, retrieval, decision, acting, post-action, and converting [26]. Images fall under the input variable ‘message’, which can be manipulated to affect the process of persuasion. This strategy allows insight in exactly where images can play a role in improving transition to the next phase of the communication/persuasion process, according to the end-users.

### Individual interviews

Participants were recruited at three distribution points of a food bank in The Hague (the Netherlands). Experienced volunteers of the food bank asked clients if they were interested to participate in the study. Clients were approached of whom volunteers suspected through previous encounters, for example, when filling out a form to register with the food bank, had difficulty reading and writing. People who expressed interest were explained the procedure by the researcher, after which informed consent was obtained. Duration of the interviews was on average 10–30 min.

To ensure that participants were indeed part of the target group, literacy levels were determined using the REALM-D, a validated instrument to measure functional literacy in health-related information in Dutch [27]. The scores of the included participants indicated they would have moderate to severe problems to understand average patient information (≤60 words correct according to the score-requirements reprinted by [28]). For mean REALM-D scores see Table 1.

**Table 1:** Demographic data of participants of individual interviews and focus group discussions

	Interviews (n = 15)	Group 1 (n = 5)	Group 2 (n = 4)	Group 3^a^ (n = 9)	Group 4 (n = 3)	Group 5 (n = 9)	Group total (n = 30)
Age							
Average	43	41	56	49	42	45	47
Median	41	39	64	55	34	41	43
Range	26–60	25–61	25–69	22–69	24–69	36–60	22–69
Sex							
Male	2	0	1	2	0	2	5
Female	13	5	3	7	3	7	25
Education level^b^							
Low	9	2	3	6	2	4	17
Secondary	6	0	0	0	0	2	2
Unknown	0	3	1	3	1	3	11
Native language							
Dutch	4	0	0	0	3	0	3
Dutch+^c^	4	2	3	7	0	1	13
Other	7	3	1	2	0	8	14
Years of speaking Dutch							
<15	2	1	0	1	0	0	2
15–30	4	3	0	3	0	8	14
≥30 years^d^	9	1	4	5	3	1	14
REALM–D score							
Average	48.5	–	–	–	–	–	
Median	55	–	–	–	–	–	
Range	0–60	–	–	–	–	–	
Reading level							
Meijerink	–	1F	1F	1F	1F	1F	
Level (local)	–	Level 2	Level 1	Level 2	Level 1	Level 2	

^a^One participant was present for both focus group 1 and 3, so that there are 44 unique ns

^b^Education levels according to Statistics Netherlands [29]

^c^This category also includes people from Surinam who only spoke Dutch at school from the age of 4

^d^This category also includes native speakers of Dutch

Sixteen people were interviewed, of which two in a joint interview. One participant was later excluded from analysis because their REALM-D score was too high to be representative for low literacy, so that the final *n* = 15. Most participants were female (*n* = 13) and the average age was 43. None of the participants were educated beyond secondary level. For eight participants, Dutch was (one of) their native language(s), while seven people indicated to have another first language.

### Focus group discussions

For the focus group sessions, participants were recruited from Dutch language classes at an educational institution for adults who are fluent in Dutch, but have a low reading and writing level (The Hague, the Netherlands). Interested candidates were explained the procedure, after which informed consent could be obtained. Group sizes ranged from three to nine participants, and depending on the size, discussions took between 30 and 60 min. The sessions were moderated by author MvB and PG and carried out with the instructor of the class present.

Literacy levels were not determined individually. According to the educational institution, all included participants had a reading level below 1 F in Meijerink’s classification, the Dutch national standard—equivalent to a reading level below that of children that finish primary school [30]. A total of 30 low-literate people took part in this part of the study, distributed across five focus group sessions. Most participants were female (*n* = 25), and the average age was 47. Most of the participants were not schooled to secondary education level. Sixteen participants had Dutch as (one of) their native language(s), 14 did not.

### Data collection and analyses

Data were collected between November 2013 and February 2014. All sessions were semi-structured and covered questions on participants’ use of written drug information, their evaluation of it, and suggestions for improvement. A randomly selected standard patient information leaflet (angiotensin-converting-enzyme inhibitor, ENA50005627-D) was shown to initiate the discussion and to make sure that the topic of ‘patient information leaflets’ more tangible.

Subsequently, the idea of adding images was discussed, followed by a discussion on possible uses of images –to facilitate the conversation, examples of images were shown, including abstract signs (blue information sign, yellow warning sign, and a red stop sign), and more informative images (USP-pictograms: “take by mouth”, “for heart problems”, “take with glass of water”, and “do not take if pregnant”) [31]. Also, a sample of a visual/textual leaflet [32] was shown, as an example of how the addition of images could look for patient information, to facilitate the discussion on possible benefits of adding images. Additional rounds were held to discuss participants’ preference concerning design style and topics for visualisation—these results require their own framework and will not be discussed here.

Audio recordings were transcribed between sessions so that data-collection could be stopped when saturation in the answers was reached. As is typical for focus group discussions, the moderators frequently summarised findings during the discussions and brought up answers that were given in previous sessions, to verify findings as they were gathered. Data were analysed using the thematic framework approach as described by Ritchie and colleagues [33]. Author MvB identified initial themes, and after independently applying this indexing framework to a subset of the interviews, authors PG and MB discussed the framework and themes in several rounds until consensus was reached. Consequently, all data were labelled and summarised using QDA Miner Lite.

## Results

Four main themes were identified in the interviews and discussions: information-seeking strategies, evaluation of written drug information, suggestions for improvement, and roles for visuals.

### Information-seeking

Most participants indicated that they normally rely on additional or alternative sources for medication instructions to the PIL (Table 2). Some refer to the medicine packaging, and a few participants said to look up words or instructions online. The majority of participants who do not read the leaflet rely on other people for their information: usually their pharmacist, general practitioner, or family members. A participant explained why she prefers this to reading written instructions: “If I do read it, then I do not understand everything. And if I get it a little wrong, I will panic completely.” Although most participants had some way to obtain their information, when asked in the focus group discussion, several participants admitted to regret not being able to gather information from the leaflet for themselves.

**Table 2:** Information-seeking strategies

Information-seeking strategies
Reading the patient information leaflet
Letting someone else read the PIL
Relying on instructions from healthcare provider (pharmacist, GP)
Seeking information on the Internet
Reading information on drug packaging

### PIL evaluation and preferences

#### Tuning in, attending and liking stages

The majority of participants did not consider PILs to be patient-friendly (Table 3). Both in the focus groups and individual interviews it was mentioned that the text was too long and the font size too small. Generally it was agreed that an ideal leaflet consists of only one A4-sized sheet that provides a clear overview of relevant information in a legible font size.

**Table 3:** Participants evaluation and preferences for PILs

Participants’ evaluation of PILs	Topics on PIL preferences
Document too long	Quantity of information/text
Difficulty of small font size	Font size
Discouraging to read	Preferred type of medium
Trouble with finding information	Organisation of information
Usefulness of headings	Clarity of leaflet
Unsuited language use	Language use
Difficult to comprehend	Use of images (in combination with text)
	Addition of an area to write in PIL

Many participants indicated that the general appearance of current leaflets was not inviting. As a participant stated: “I simply do not feel like reading it when I see it like that.” Those who read the leaflet often indicated that it is difficult to work up enough motivation to read and process the information in the leaflet, particularly if they feel unwell and need to take the medication quickly. Similarly, most participants said to prefer printed over digital medication instructions, so that it is within easy reach. Also, a few participants did not have regular access to a computer, so that providing digital information alone would not suffice.

Finding relevant information in the leaflet was considered too difficult by the majority. A participant explained that “it is only in the middle and at the end that you can find what is really important.” Regularly, the usefulness of headings was mentioned to navigate the text: “I always first look at the heading, the title of a section, and if this seems to be information I need than I will read that section of the text.”

#### Comprehending, generating, acquiring stages

Most participants agreed that the patient leaflet is difficult to read. A few participants mentioned their own reading level in this context, but the unnecessary use of complex language was considered the biggest problem. This was illustrated by a participant’s comment: “The information is written in Latin doctor language. It is the language of the pharmacy, words of the general practitioner - not of ordinary people.” In addition to the use of simple language, it was suggested to provide information in the user’s native language.

During the focus group discussions, participants came up with the idea to add an area where people can take notes, for example on when to take the next dose. When the subject of visuals as a supplement to text was discussed, some participants expressed that for them it was not necessary, but the majority was enthusiastic – especially to use images in combination with text.

### Roles for visuals

#### Tuning in, attending, liking stages

Participants saw a variety of uses for images (Table 4). Visuals were thought to make the leaflet more appealing, less dull and daunting, and thereby more inviting to use. Leaflets with visuals were expected to draw more attention than leaflets without visuals. One participant stated: “People generally think ‘I do not want to read the leaflet’, so [adding visuals] makes [the leaflet] more fun to read.”

**Table 4:** Roles for visuals in the PIL

Uses for visuals in PILs as identified by low-literate participants
Make the leaflet look more *appealing* and *inviting*
Help *navigate* the leaflet to find relevant information
Help to *highlight warnings*
Serve as a tool to *ask questions* to caregivers
*Explain* and help *understand* what is written in text
Provide an *overview* of the information in the leaflet
Help to *recall* information
*Preview* what needs to be done in a visual instruction
*Reassure care*-*receivers* by showing what they can expect
Contribute to a *clearer, easier* and *quicker* to use, *shorter* leaflet
Enhance a feeling of *confidence, empowerment* and *safety*

In addition, participants thought that visuals could draw attention to specific topics. By serving as a cue, a visual could prove useful by highlighting topics that should not be overlooked, including warnings. This way of using images was also considered helpful by participants whose reading levels do not allow them to read the text themselves, and who could use visual cues to ask others to help read them particular sections of text.

#### Comprehending, generating, acquiring stages

Quite a few participants also thought that more informative images, such as pictograms, could provide a visual overview of the leaflet to help get a first impression of the information, and enable them to navigate the text and read selectively. Pictograms could also help to understand or even eliminate the need to read written information. This was thought to make information in the leaflet quicker to retrieve, clearer and easier to understand - especially since some participants indicated that it is easier to extract meaning from a visual than from a text.

According to a few participants, visuals could be used to validate their understanding of text and could empower and increase confidence. In this context, it was considered helpful to have visuals that show required actions, for example, a pictogram of how the medicine should be administered. It was suggested by a participant that such visuals could also be used to reassure children by showing what will happen. Yet another participant mentioned that adding visuals would make her feel safer, again in the context of children and medication.

#### Retrieval stage

Some participants also saw a role for pictograms in recalling previously acquired knowledge: “Then you would not have to read the leaflet in its entirety, you would have this visual language.” Many participants were confident in their ability to recall verbal information provided by their pharmacist, but considered it useful to have visuals for cued recognition. This was especially the case for people who were mostly reliant on verbal information, because “there is always a chance that you forget what they have said.”

## Discussion

The aim of this study was to gain insight into how people with low literacy evaluate written drug information, and to identify ways in which images could help them improve this experience. We found that individuals with low literacy are discouraged by the overall look of patient information, as well as by its content, as relevant information is difficult to find and to understand. These findings are concurrent with previous research concerning patients’ experience with drug information [2, 10, 34]. Although a printed leaflet is still the preferred medium to receive medication instructions, existing materials are unsuited, which makes patients with low literacy reliant on other people, drug labels, or on online sources of varying reliability.

People have a limited capacity for processing new information, and different channels for processing words and pictures [35]. Low-literate people indicate that they experience a high cognitive load when required to read written drug information, which is reflected in their comments about the time and effort it takes to read and process the information - visuals may help to limit this load [36].

Low-literate people find visuals to be a useful supplement for a variety of reasons. Images can provide a quick overview of and context to the information in a leaflet, and can facilitate understanding of what is written in text. In the context of McGuire’s model of mass communication (Table 5), and the corresponding stages that people proceed through to reach successful persuasion, this translates to that images can help to progress through the ‘comprehending’ phase (4). Several studies have shown that images, and especially pictograms, can be an effective tool to increase patients’ understanding of textual information [13, 18, 37]. In particular, low-literate people are interested in images that show exactly what they have to do, to facilitate the generation of the cognitions (5) and acquire the skills (6) necessary to take their medication as prescribed.

**Table 5:** Communication outputs that can be targeted by visuals

Outputs as described by McGuire	Illustration of a persuasive process to adhere to medication, taking place through the patient leaflet.
(1) Tuning in	Exposure to the PIL
(2) Attending	Paying attention to the PIL
(3) Liking	Liking, maintaining interest in the PIL
(4) Comprehending	Understanding the message in the PIL eg., “I should take my medication every day”
(5) Generating	“I know what I can do to make sure I take my medication every day”
(6) Acquiring	“I know how I can take my medication every day”
(7) Agreeing	“I agree it would be good to take my medication as prescribed”
(8) Storing	“I have stored in my memory that I want to take the medication every day”
(9) Retrieval	“I remember this at relevant times”
(10) Decision	“I am going to take my medication every day”
(11) Acting	“I really do take my medication every day”
(12) Post-action	“I have integrated taking my medication into my life”
(13) Converting	“Others should also take their medication as prescribed”

In addition to enhance central processing, many suggestions relate to the idea of visuals as peripheral cues (e.g., to make the leaflet more appealing, or to attract attention to the leaflet or particular topics) [38, 39]. In this role, images can help the patient to successfully proceed from the tuning in phase (1), to attending (2) and liking (3) - essential to reach the comprehending (4) stage. This is important given the finding that many people with low literacy find it difficult to work up the motivation required for central processing, or to ‘decipher’ the information. As peripheral cues, images also increase the likelihood of people with low elaboration to be persuaded by and agree (7) with the message, and decide (10) to act upon it (11), without central processing taking place [38, 39].

This, however, does not mean that purely decorative images need to be added to patient information, as previous research has shown that this can be experienced as distracting [40]. It is likely that appeal of the document can be increased enough by providing structure and support in the form of explanatory images that increase the readability of the text and images that highlight important topics.

The latter, ‘flagging’ information, is an important potential role for images, as one of the biggest concerns of the participants was how difficult it is to find relevant information in the leaflet – exactly why they were so appreciative of headings in the text, a finding that is supported by previous studies [41, 42]. In addition, one of the ways in which low-literate people said to obtain information on their medicine was to ask a caregiver or family member to read the PIL for them. In this way, images that highlight can work empowering, by allowing patients who cannot read to ask for specific information from their healthcare provider or caregiver based on what they can see in the pictogram.

Low-literate people also see a role for images to help remember information (9) that has been communicated previously. Many are dependent on information they receive orally, and visual confirmation can help to enhance their confidence in the knowledge they have.

Generalisations should be made with care, given the qualitative nature of this study, the convenience sampling, and the fact that the majority of the sample was female. Social stigmas [43] may prevent participants from being completely open about difficulties they experience. However, the instructor’s presence during the focus group sessions encouraged participants to openly contribute to the discussion. Even if participants were somewhat reserved in their answers, the results will underestimate the problem rather than overestimate it, and it is expected to have been of little influence on the way people responded about possible uses for visuals. Another limitation of the study is that in order to make the topics under discussion more approachable, it was necessary to show examples of both leaflets and visuals. The selection of these materials may have influenced how participants viewed the topic, so that they did not have full creative freedom in their answers. However, in every session it was attempted to start the conversation as open as possible, so that the authors feel that showing the examples was a necessary step to successfully engage in a participatory design process with low-literate end users.

## Conclusions

People with low literacy experience barriers in communication via written drug information early on in the communication/persuasion process, i.e., at the stages of paying attention to the leaflet and maintaining interest in the message. These barriers can be lowered through the use of short, structured textual explanations, supported by images. This approach further has the potential to improve understanding of information and to empower the target group of low-literate patients. The outcomes of this study confirm the roles for images as described by EU-regulations on patient leaflets, i.e. images to highlight topics, aid navigation and clarity text (Article 62 of Directive 2001/83/EC), and suggest an equally important role for images to lower the motivational threshold for patients with low literacy to take interest in the information. By honouring the input of the target group in this very early stage of the design process, a resulting visual/textual intervention is more likely to match the information preferences and needs of people with low literacy, and may even have the empowering effect that participants of this study themselves describe.
